# *Helicobacter pylori* and Non-*Helicobacter pylori Helicobacter* (NHPH) Zoonotic Infections: A Survey Among Greek Veterinarians Aiming to Enhance Communication

**DOI:** 10.3390/pathogens15020227

**Published:** 2026-02-18

**Authors:** Eirini Fragkiadaki, Francisco Cortez Nunes, Maria Linou, Beatriz Martinez-Gonzalez, Dionyssios N. Sgouras, Teresa Letra Mateus

**Affiliations:** 1 Department of Animals Models for Biomedical Research, Hellenic Pasteur Institute, 11521 Athens, Greece; 2 Faculty of Medicine and Biomedical Sciences, University of Algarve, 8005-139 Faro, Portugal; 3 Hellenic Veterinary Medical Society (HVMS), 158 Patision Street, 11257 Athens, Greece; president@hvms.gr; 4 Laboratory of Medical Microbiology, Hellenic Pasteur Institute, 11521 Athens, Greece; bmartinez@pasteur.gr (B.M.-G.); sgouras@pasteur.gr (D.N.S.); 5 CISAS—Centre for Research and Development in Agrifood Systems and Sustainability, Escola Superior Agrária, Instituto Politécnico de Viana do Castelo, 4900-347 Viana do Castelo, Portugal; tlmateus@esa.ipvc.pt; 6 Unidade de Investigação em Epidemiologia (EPIUnit)—Instituto de Saúde Pública da Universidade do Porto, Laboratory for Integrative and Translational Research in Population Health (ITR), Rua das Taipas, nº 135, 4050-091 Porto, Portugal; 7 Veterinary and Animal Research Centre (CECAV), Associate Laboratory for Animal and Veterinary Sciences (AL4AnimalS), University of Trás-os-Montes and Alto Douro (UTAD), Quinta de Prados, 5000-801 Vila Real, Portugal

**Keywords:** *Helicobacter* spp., One Health, zoonosis, science communication, occupational risk, awareness

## Abstract

*Helicobacter* species affect humans and animals, mainly causing gastrointestinal but also extra-gastrointestinal pathologies. Besides *Helicobacter pylori*, which is the main human pathogen, Non-*Helicobacter pylori Helicobacters* (NHPH) are also associated with human diseases, thus raising concern about their zoonotic potential. Veterinarians are considered a risk group for NHPH infections and act as first-line communicators to animal owners about their prophylaxis. Therefore, we aimed to assess the knowledge and perception of veterinarians working in Greece about *Helicobacter pylori* and NHPH by asking them to participate anonymously in an online 34-question survey. The questionnaire consisted of three sections regarding environmental exposure to *Helicobacter* spp.; know-how about clinical signs in various species, including personal human experience; and willingness to get updated information about NHPH. Of the 111 respondents, 41.4% had not heard of *H. suis* (NHPH), and 35.0% were unaware of the species that could be affected. Almost 60.0% of companion animal veterinarians rarely suspect and 20.0% never suspect *Helicobacter* spp. infections in the case of gastritis. Nevertheless, 41.0% of respondents considered *Helicobacter* as zoonotic, and 87.0% wanted to receive information via professional channels and brochures. Despite the limited number of respondents and the exploratory nature of our study, as with similar data from Portugal, we emphasize the need to train veterinarians to have a more targeted focus on the zoonotic potential of *Helicobacter* within a One Health approach.

## 1. Introduction

The genus *Helicobacter* comprises Gram-negative, spiral or curved, motile, microaerophilic bacteria that colonize the gastrointestinal tract, particularly the stomach of humans and animals [1,2,3]. Based on their ecological niche, these organisms are classified into two main groups: gastric and enterohepatic species. Among gastric species, *Helicobacter pylori* is the most clinically important. It infects the human stomach, is strongly linked to gastritis and peptic ulcers, and has been determined to be a major risk factor in the development of mucosa-associated lymphoid tissue (MALT) lymphoma and gastric cancer [4,5,6,7,8,9]. The global prevalence of *H. pylori* infection is estimated to be approximately 43%, with a declining trend reported worldwide, particularly in the last decade [10,11]. Unlike *H. pylori*, other members of the genus, collectively referred to as non-*Helicobacter pylori Helicobacter* (NHPH), have animals as their natural host but are also capable of infecting humans, in whom they have also been associated with gastrointestinal diseases [12]. Numerous studies have highlighted the clinical relevance of NHPH infections in both humans and animals, including farm, companion, and wild animals, which serve as natural reservoirs. Multiple different NHPH species have been isolated from the gastric mucosa of a broad range of mammals, including dogs, cats, ferrets, pigs, monkeys, and cheetahs [5], where they have been associated with varying degrees of gastritis and represent a potential source of zoonotic infection [12,13]. Transmission to humans is thought to occur primarily through close contact with infected animals or exposure to contaminated animal-derived products. Most clinically relevant gastric NHPH species, including *H. suis*, *H. felis*, *H. bizzozeronii*, *H. salomonis*, *H. ailurogastricus* and *H. heilmannii*, are increasingly recognized as zoonotic pathogens and have been linked to similar gastric human diseases, including gastritis [14], gastroduodenal ulcer disease [15] and gastric cancer [13,16]. Many of these Helicobacters have also been associated with diarrhea, bacteremia and systemic disease in immunocompromised hosts [17]. Importantly, several of these species, particularly *H. heilmannii*, *H. felis*, *H. bizzozeronii*, and *H. suis*, exhibit a credible zoonotic potential, particularly in the context of veterinary risk assessment, where close contact with infected dogs, cats, pigs and primates or exposure to contaminated animal-derived products increases the likelihood of transmission [18]. Indeed, the zoonotic relevance of NHPH remains underappreciated due to the lack of large-scale epidemiological studies that limit accurate assessments of transmission routes and public health impact [19]. The global prevalence of NHPH infection in humans during the 2010s remained significantly lower than that of *H. pylori*, with percentages ranging from 0.1% to 6.2% depending on the geographic location, the specific study group and detection methods used [12,20,21]. Indeed, the incidence of NHPH infection was higher in studies using molecular techniques. Notably, recent studies suggest that NHPH prevalence in human gastric samples ranges broadly from approximately 20% to 30% in selected symptomatic patients [17,20]. In particular, Taillieu et al. (2023) [19] reported a prevalence of gastric NHPHs of 29.1%, highlighting that these organisms were not rare incidental findings but rather a substantial component of the gastric microbiota in the study population. Preliminary observations from symptomatic Greek patients suggested marked variability in NHPH prevalence, ranging from 0.9% to 29.4% [22]. This heterogeneity was strongly influenced by patient age and the specific *Helicobacter* species detected. Furthermore, in a recent Portuguese cohort using molecular PCR analysis, 35.9% of patients were positive for *H. bizzozeronii*, representing a high local prevalence of this specific NHPH species [23]. In addition to gastric species, other NHPH members colonize the intestine, biliary tree and liver of animals. Notably, the presence of *H. cinaedi* and *H. bilis*, the most prominent enterohepatic NHPH species, has been associated with invasive human infections, including cellulitis, sepsis, osteomyelitis, and pyoderma gangrenosum [24]. Sporadic human cases involving other enterohepatic species, including *H. fennelliae*, *H. canis*, *H. equorum*, *H. canadiensis* and *H. pollurum*, have also been reported [25,26,27]. All these findings underscore the growing recognition of NHPH species in human gastric pathology. Accurate detection is essential not only for understanding the epidemiology of these emerging zoonotic pathogens but also for preventing their transmission and implementing effective control measures. Eradication of these NHPH species through standard antimicrobial therapy, typically the same regimens used for *H. pylori* eradication, has also resulted in the resolution of gastritis, peptic ulcer disease and clinical remission of MALT lymphoma [28].

From a One Health perspective, integrating human and animal surveillance is critical to managing the risks posed by NHPH at the human–animal nexus. Veterinarians are considered a high-risk group for NHPH infections and play a crucial role as first-line communicators advising animal owners on preventing and prophylaxis measures. This exploratory study aimed to assess the knowledge and perceptions of veterinarians working in Greece regarding *H. pylori* and NHPH infections through an anonymous online questionnaire survey.

## 2. Materials and Methods

### 2.1. Data Collection

Data collection was performed using a structured questionnaire originally developed and content-validated in [29] through bibliographic review, focus groups with veterinarians, and pilot testing. The questionnaire was translated and adapted into Greek for the purposes of the present study. The questionnaire consisted of three sections: one related to general demographic information of the participating veterinarians, the characteristics of contact with pigs and wild boars, referring to duration and type of contact (physical or via food consumption) and the hygiene habits of participants during food preparation. The second section investigated the perceptions and knowledge about *Helicobacter* spp. infections either for *H. pylori* or NHPH, posing questions about (a) their animal hosts, the clinical signs and the NHPH inclusion in the differential diagnosis of digestive pathologies in companion animals’ practice; as well as (b) the anamnesis of participants’ gastric problems, their diagnostic investigation and applied pharmacological regimen and, finally, a specific question whether they were affected by *H. pylori* and if it was successfully treated. Finally, in the third section, after introducing the animal–human transmission potential of NHPH, questions regarding the participants’ interest in receiving more information are posed and which communication channels they prefer best for the dissemination of such One Health concepts (i.e., via SMS/calls, social media, leaflets-brochures, family doctors, Hellenic Veterinary Association-HVA, Ministry of Agriculture and Food, occupational doctors, nurses) are addressed.

Thirty-four dichotomic, multiple-choice, rating scale, matrix, drop-down and open-ended questions composed the final survey, which took around 15 min to complete. Respondents could choose not to answer questions and could exit the survey whenever they wished. Therefore, the number of received data points is not constant per question.

### 2.2. Study Design and Participants

The questionnaire was disseminated among certified veterinarians practicing in Greece. A virtual snowball sampling survey was run first through e-mail to the official mailing list of Hellenic Veterinary Medical Society (official veterinarians’ scientific society) and then using closed social networking channels (namely, Facebook Messenger, Meta Platforms, Menlo Park, City of California, CA, USA) via personal invitation to work colleagues, veterinarian friends and classmates. It was not possible to distinguish the two sources of participation from the answers received. The minimum sample size was calculated to be 366 when using Yamane’s Simplified Formula [30] for the sample on n = N/1 + N × E^2^, where N is the population size (namely, 4400 veterinarians based on the Geotechnical Chamber of Greece-GCG registry, 2024). E is the level of precision, which was set to 5% or 0.05. However, the achieved sample of 111 respondents was lower than the calculated target. Consequently, this study should be interpreted as an exploratory, hypothesis-generating survey rather than a statistically powered investigation. The findings are therefore not intended to be representative of the national veterinary population but to provide preliminary insights into awareness patterns and educational needs.

The survey was active in two periods from September–November 2024 to August–October 2025. Receiving duplicate responses was quite unlikely, given the participants’ integrity and the limited availability to participate once, let alone repeat the survey.

### 2.3. Ethical Approval and Consent to Participate

The Research Ethics and Deontology Committee of Hellenic Pasteur Institute reviewed the research team’s application for the project, ensuring that it is in compliance with European and national legislation regarding Ethics and Data protection of the involved human individuals. The project was unanimously approved (decision n. 4952 issued on 8 August 2024). All participants were informed about the use of collected information, and their consent was mandatory by selecting the relevant box at the very beginning of the survey in order to proceed to the questions. No data leading to the identification of the participants were collected.

### 2.4. Statistical Analysis

Statistical analysis was performed using IBM SPSS^®^ Statistics v27 (IBM, Armonk, NU, USA). Descriptive statistics were the initial stage of data presentation. Possible associations between nominal variables were performed using cross-tables and chi-square (with *p* < 0.05) [30].

## 3. Results

### 3.1. Demographic Description of Respondents

More female veterinarians participated in the survey (women 57.7%, 64/111, 25 to 73 years old, mean age 46.3 ± 11.8; men 40.5%, 45/111, 25 to 87 years old, mean age 49.4 ± 12.7; not answered 2/111), with 67.5% (75/111) of the total respondents being companion animal veterinarians and at an overall mean age of 47.6 ± 12.2 years old.

**Figure 1:**
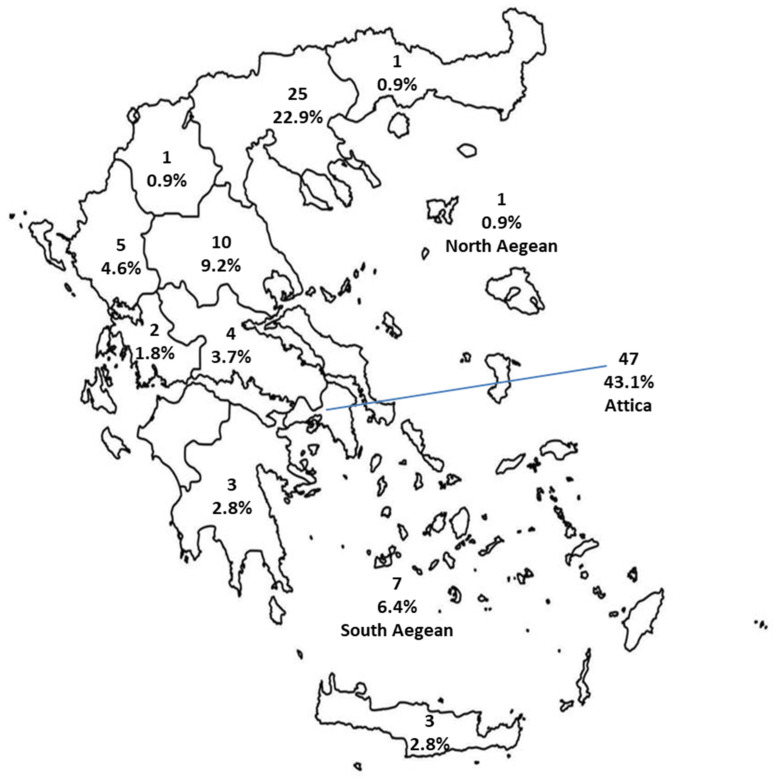
Geographical distribution of respondents, depicting their number and percentage per administrative region (i.e., Attica 47/109, 43.1%). (The island groups of the North and South Aegean Sea and Attica are indicated on the map for clearer graphical representation to the reader).

Respondents worked in 12 of the 14 administrative regions of Greece, with a greater percentage located in Attica (43.1%), Central Macedonia (22.9%) and Thessaly (9.2%) (Figure 1, Vecteezy map adapted to data).

### 3.2. Awareness of Environmental Exposure

#### 3.2.1. Physical and Nutritional Contact with Swine

Regarding physical exposure to swine, 66.7% (74/111) of the veterinarians replied that they had rare (56/111) and frequent (18/111) contact with live pigs or wild boars. Notably, 29.7% (22/74) of veterinarians contacted them via livestock animal practice, 17.6% (13/74) via companion animal practice, 20.3% (15/74) as meat inspectors, 29.7% (22/74) in other circumstances (hunting, coexistence in the habitat) and just 2.7% (2/74) as livestock farmers. The duration of contact was up to 5 years among 66.2% (49/74) of veterinarians, and 33.8% reported longer durations.

Data about dietary exposure to swine meat and meat products revealed that 94.6% (105/111) consume pork meat and 86.5% (96/111) consume pork products, while the percentages for wild boar meat and products are 29.6% (44/111) and 19.8% (22/111), respectively.

#### 3.2.2. Food Safety and Hygiene Practices

Just around 9.0% (10/110) of veterinarians mentioned using borehole water for drinking and food preparation. On the other hand, 96.4% (107/110) reported that they prepare their own meals, with over 90% consuming them more than 3 times per week.

Of the 109 veterinarians who prepare their own meals at home, 89.0% (97/109) stated that they have hygiene and safety measures taken during food preparation, 9.2% (10/109) had no such measures, just 1 of them (1/109) was unaware of them and another one preferred not to say. The hygiene measures undertaken were stated to be washing of raw materials (36.9%, 41/111), meat and vegetables separation to prevent cross-contamination (28.8%, 32/111), disinfection of surfaces used for food preparation (10.8%, 12/111), hand washing (11.7%, 13/111), well-cooked meals (9.9%, 11/111) and use of gloves (7.2%, 8/111).

### 3.3. Know-How About Helicobacter *spp.* Infections

#### 3.3.1. Animal Hosts and Clinical Signs

All respondents had heard about Helicobacter, with 9.0% (10/111) not knowing accurately what it is. On the other hand, 3.6% (4/111) and 41.4% (46/111) had never heard of *H. pylori* and *H. suis*, respectively, while the percentage of not accurately knowing about them was 8.1% (9/111) and 28.8% (32/111), respectively (Table 1).

Regarding the open question, “which animal species can be affected by *Helicobacter* spp.”, the majority replied dog (38.7%, 43/111), pig (36.9%, 41/111), cat (21.6%, 24/111), human (19.8%, 22/111) and primates (1.8%, 2/111), while 35.0% (39/111) gave an ambiguous-no answer or do-not-know reply. Regarding the clinical signs of Helicobacter infections in animals and humans only, 7.2% (8/111) replied that they do not know.

The majority of companion animal veterinarians replied that they never (20.0%, 15/75) or rarely (60.0%, 45/75) include *Helicobacter* spp. infection in the differential diagnosis of gastritis in dogs and cats, with just 4.0% (3/75) always and 16.0% (12/75) frequently replying to consider it. The low suspicion rate for Helicobacter infections may contribute to underdiagnosed cases or delayed therapeutic interventions targeting these pathogens. Interestingly, 14.8% (9/61) stated that they had a confirmed diagnosis of small animals’ gastritis due to the presence of Helicobacters.

Regarding the human–animal transmission of *Helicobacter* spp., 41.0% (43/106) considered it a zoonotic pathogen, 16.0% (17/106) replied negatively and 43.0% (46/106) stated that they did not know.

#### 3.3.2. Anamnesis of Gastric Pathologies Among Veterinarians

From the total number of respondents, 50.0% (55/109) had rarely and frequently experienced gastric pain, 18.0% (20/110) had been diagnosed with gastritis, 24.0% (26/109) had often or everyday gastric reflux or heartburn and 37.0% (41/110) had laboratory tests for gastric pathologies mainly via endoscopy (Table 2). Regarding treatment, 30.0% had received medication for gastric pathologies, referring mainly to proton pump inhibitors like omeprazole.

Additionally, 15.0% (16/107) of veterinarians had gastric problems associated with *H. pylori*. Fifteen veterinarians received specific treatment for *Helicobacter* infection, and all stated that the specific treatment was successful.

### 3.4. Willingness to Learn and Preferred Channels of Communication

After presenting in a brief paragraph the zoonotic potential of NHPH, we asked if participants wished to receive more information about the topic. The majority were positive to learn more (87.3%, 96/110), and the three most preferred dissemination channels were, in descending order, through the Hellenic Veterinary Association (HVA, 68.0%), the Ministry of Agriculture and Food (56.0%), and via leaflets and brochures in their professional location (48.0%) (Table 2).

### 3.5. Statistical Associations Between Variables

After recoding the variables into dichotomous yes/no categories, cross-tabulations and chi-square tests for the 2 × 2 tables were conducted. No statistically significant associations were identified between the variables.

## 4. Discussion

The significance of *H. pylori* infection is well recognized globally for its impact on both benign and malignant gastric disease in humans. Other NHPH species, like *H. suis*, *H. felis* and *H. bizzozeronii*, which are prevalent in animals, have been associated with dyspepsia, epigastric pain, acid reflux or gastric MALT lymphoma in humans, thus providing evidence of their animal–human transmission [19].

Taking into consideration the rising concern of NHPH species in public health, the contact of humans with farm and mainly pet animals, the role of food in the transmission of *Helicobacters* [31] and the case of *H. suis* infection in a veterinarian [32], we wondered about the preventive role of veterinarians against these pathogens. A recent literature search revealed limited veterinary data, with most studies referring to the detection of *H. pylori* in raw cow milk [33] and the development of a pig model for *H. pylori* [34] from Greek research teams. No published data implied the perception of Greek veterinarians about *Helicobacter* infections and their One Health implications. Our aim was to investigate, for the first time, our veterinary colleagues’ awareness of *Helicobacter* infections and their risk factors for exposure, and to identify their preferred channels of communication for receiving scientific information. For this purpose, we adapted the questionnaire targeting Portuguese veterinarians [29], also considering the similar socioeconomical conditions and population size of both countries.

This study should be interpreted as an exploratory pilot investigation. The limited sample size and non-probabilistic sampling strategy do not allow for population-level generalizations, but the findings provide initial insights into awareness patterns and educational needs among participating veterinarians. On the other hand, it adds more evidence from similar stakeholders regarding the topic [29], compared to studies from the general population, as performed in Bosnia and Herzegovina [35].

In our survey, a higher proportion of female veterinarians participated (57.7%, 25 to 73 years old, mean age 46.3 ± 11.8) compared to male veterinarians (40.5%, 25 to 87 years old, mean age 49.4 ± 12.7). In 2024, 42.21% female and 57.79% male veterinarians were registered in GCG, while in 2023, according to the Federation of Veterinarians in Europe (FVE), 65.0% of veterinarians working in EU countries were female [36]. In all cases, no association among demographic characteristics and study variables was observed.

Of the 111 respondents, direct exposure to live swine was present (66.7%), but the most usual exposure was by food consumption, since 94.6% consumed pork meat and 29.6% consumed wild boar meat, plus their meat products in a smaller percentage. This observation provides contextual information relevant to previously reported transmission routes, given the reported transmission of *H. pylori* via the fecal–oral route and contaminated food ingestion [31] and *H. suis* presence in pig carcasses and retail meat [37,38]. The respondents preferred to prepare meals at home (96.4%) at least three times per week, and the vast majority (86.0%) took hygiene and food safety measures during the process. Nevertheless, the gold standard "hand washing" was reported much less frequently (11.7%), similar to the Portuguese survey [29], compared with washing of raw materials (36.9%) and separating meat and vegetables (28.8%) to prevent cross-contamination. Fortunately, the potentially contaminated borehole water was used only by 9.0% of respondents, not constituting a common risk factor in our sample.

Regarding the infectivity of *Helicobacter*, 41.4% of the veterinarians had never heard of *H. suis*, and 28.8% did not know exactly what it was, which confirmed our hypothesis about limited NHPH information. About 35.0% of the respondents were unclear or unaware about the animal species affected by *Helicobacter*, and 60.0% of companion animal veterinarians rarely considered it in the case of gastritis, which made us reflect on their alertness in suspecting the pathogen. On the other hand, just 4.0% of the companion animal veterinarians always thought of *Helicobacter* infection in case of gastritis in dogs and cats, and 14.8% affirmed its presence microbiologically. And even though all veterinarians had heard of *H. pylori*, its zoonotic potential seemed not to be clearly perceived, as 43.0% stated that they did not know if *Helicobacter* infections are zoonotic, and 16.0% stated they are not.

Even though half of the veterinarians had occasionally experienced gastric pain, 30.0% had received mainly proton pump inhibitors to alleviate gastric discomfort. Of the 107 respondents, 15.0% had gastric problems due to *Helicobacter* and underwent specific treatment that was fully successful. However, no correlation is statistically justifiable about their veterinary specialization, years of contact with live animals or gender that allows us to elaborate about their linkage.

It became evident that Greek veterinarians have limited knowledge about *Helicobacter* infections and uncertainty about their human–animal transmission, just like their colleagues from Portugal 29]. The Portuguese survey revealed that 37.2% of the respondents had not heard of *H. suis*, 17.6% were unaware of the animal species that could be affected and 76.2% did not consider Helicobacter infection in the differential diagnosis of gastritis (compared to, respectively 41.4%, 35.0% of Greek respondents and 80% that never or rarely considered *Helicobacter* in gastritis cases). Interestingly, 47.7% of Portuguese respondents and 41.0% of Greek respondents considered *Helicobacter* spp. a zoonotic bacterium. The understanding of one target group’s risk perception (veterinarians in the present study) is essential to be able to assess communication needs and messages to be delivered in the future [39]. An encouraging finding is that 87.3% of the respondents wished to learn more about the topic and mainly indicated official channels of information (like the HVA and Ministry), probably as more reliable contact institutions. Brochures were the third most preferred way of communication compared to SMS or social media posts, maybe due to the mean age of respondents, which was quite higher compared to the digitally adapted Generation Z. Some veterinarians (9.1%) wished no further communication, which is respected, although not fully understandable.

The microbial interconnections between humans and animals [40] emphasize the need for collaboration between medical doctors, veterinarians and environmental professionals, and high-precision diagnostic services, especially due to the climate crisis and emerging zoonotic diseases. The application of the One Health approach is only achieved if there is frequent communication across the sectors involved—in the present case, veterinarians and human doctors—and for that purpose, it needs to be formalized in order to, for example, create epidemiological data that can be shared [39], which regrettably rarely happens nowadays. Awareness regarding epidemiology and diagnosis is feasible through targeted training modules, infographics in social media, brochures distributed at veterinary/medical clinics and interactive sessions in veterinary and human medicine and nursing congresses regarding these bacteria. The predictions for the next five years, based on the Federation of Veterinarians in Europe (FVE), indicate a growing demand for veterinary services in companion and exotic animals, along with a strong demand for training in communication, business and digital skills [36]. Veterinarians are first-line health professionals against zoonotic diseases, and their preparedness in disseminating scientific information, in a precise and simple way, to animal owners is crucial.

## 5. Conclusions

This exploratory study presented the limited up-to-date knowledge of participating veterinarians about *Helicobacter* infections, particularly about NHPH, given that 41.4% have not heard about *H. suis* and 43.0% stated that they did not know if *Helicobacter* infections are zoonotic. On the other hand, although the sample size does not allow us to draw statistical inferences about the veterinarians’ population, *Helicobacter* infections may be underdiagnosed, as 80.0% of companion animal veterinarians reported never or rarely suspecting *Helicobacter* spp. infections in the case of gastritis in companion animals.

The sensitization of most respondents to the *Helicobacter* infections was practically expressed through their declared willingness to receive more information and by indicating to us the most effective communication channels for them. Additionally, the interest of 32.4% respondents, expressed in the free comments area of the questionnaire, to receive the outcomes of the survey and more information regarding *H. pylori* and NHPH was a noticeable success of our first attempt to enhance communication.

The need for continuous professional education on zoonotic diseases is paramount to train both medical doctors and veterinarians to collaborate efficiently and create synergies for the benefit of One Health protection.

## Figures and Tables

**Table 1:** Knowledge of respondents about *Helicobacter* spp. infections; questions and answers regarding number (N) and percentage (%).

Question	N	Answer	%
Have you heard about *Helicobacter* spp. infections?	111	Yes, aware of it	91.0
		Yes but not sure	9.0
Have you heard about *Helicobacter suis*?	111	No	41.4
		Yes, aware of it	29.7
		Yes but not sure	28.8
Have you heard about *Helicobacter pylori*?	111	No	3.6
		Yes, aware of it	88.3
		Yes but not sure	8.1
Which animals are infected by *Helicobacter* spp.? (open question with more than one answers accepted)	111	Dog	38.7
		Pigs	36.9
		Cat	21.6
		Human	19.8
		Non-human primates	1.8
		Ambiguous answer	21.6
		Do not know	13.5
What are the symptoms of *Helicobacter* spp. infections in animals?	111	Incorrect answers	14.4
		Correct answers	78.4
		Do not know	7.2
As a companion animals’ veterinarian, do you consider *Helicobacter* spp. as a differential diagnosis of gastritis?	75	Never	20.0
		Rarely	60.0
		Frequently	16.0
		Always	4.0
As a companion animals’ veterinarian, have you diagnosed gastritis from *Helicobacter* spp.?	61	No	85.2
		Yes	14.8
Do you consider *Helicobacter* infections as a human–animal transmissible disease?	106	No	16.0
		Yes	41.0
		Do not know	43.0
Would you like to receive more information about *Helicobacter* infections in companion animals, livestock animals, and humans?	110	Yes	87.3
		No	9.1
		I do not know	3.6
Best channel indicated to receive this type of information? (each veterinarian had up to 3 selections, and the results are presented cumulative per selection, expressed as percentage to total 111 participants)	75	Hellenic Veterinary Association	68.0
	62	Ministry of Agriculture and Food	56.0
	53	Leaflets/brochures	48.0
	43	Family doctor	39.0
	38	Social media	34.0
	19	SMS/calls	17.0
	15	Occupational doctor	14.0
	7	Nurse	6.0

**Table 2:** Assessment of veterinarians’ gastric pathology: questions and answers in numbers (N) and percentages (%).

Question	N	Answer	%
Did you used to have gastric pain?	109	Never	50.0
		Rarely	39.0
		Frequently	12.0
Have you been diagnosed with gastritis?	110	No	75.0
		Yes	18.0
		I do not know	6.0
Do you have gastric reflux/heartburn?	109	Never	41.0
		Rarely	35.0
		Frequently	20.0
		Always	4.0
Have you done any gastric diagnostic tests?	110	No	63.0
		Yes	37.0
If yes, what diagnostic test was performed?	40	Endoscopy only	73.0
		Endoscopy with biopsy	25.0
		Ambiguous answer	2.0
Do you take any medication for gastric problems?	108	No	69.0
		Yes	30.0
		I do not know	1.0
If yes, which type of medication?	32	Proton Pump Inhibitors (i.e., omeprazole, pantoprazole)	90.6
		Antacid drugs	9.4
Have you ever had any gastric problems associated with *Helicobacter*?	107	No	72.0
		Yes	15.0
		I do not know	13.0
Have you received treatment for *Helicobacter* infection?	46	No	67.4
		Yes	32.6
If yes, was the infection successfully treated?	14	Yes	100.0

## Data Availability

The original contributions presented in this study are included in the article. Further inquiries can be directed to the corresponding authors.

## Abbreviations

The following abbreviations are used in this manuscript:

NHPH	Non-*Helicobacter pylori Helicobacter*.
HVA	Hellenic Veterinary Association.
NGC	National Geotechnical Chamber.

## References

[1] Marshall B.J., Warren J.R. (1984). Unidentified curved bacilli in the stomach of patients with gastritis and peptic ulceration. Lancet.

[2] Kroiß M., Teodorescu B., Song T., Vasapolli R. (2024). Review of non-*Helicobacter pylori* Helicobacter species: Insights into pathogenesis, epidemiology, and clinical implications. Microb. Health Dis..

[3] Ménard A., Péré-Védrenne C., Haesebrouck F., Flahou B. (2014). Gastric and enterohepatic helicobacters other than *Helicobacter pylori*. Helicobacter.

[4] Malfertheiner P., Megraud F., O’Morain C., Bazzoli F., El-Omar E., Graham D., Hunt R., Rokkas T., Vakil N., Kuipers E.J. (2007). Current concepts in the management of *Helicobacter pylori* infection: The Maastricht III Consensus Report. Gut.

[5] Wang C., Yuan Y., Hunt R.H. (2007). The association between *Helicobacter pylori* infection and early gastric cancer: A meta-analysis. Am. J. Gastroenterol..

[6] Sgouras D., Tegtmeyer N., Wessler S., Kamiya S., Backert S. (2019). Activity and Functional Importance of *Helicobacter pylori* Virulence Factors. Helicobacter pylori in Human Diseases.

[7] Mégraud F., Bessède E., Varon C. (2015). *Helicobacter pylori* infection and gastric carcinoma. Clin. Microbiol. Infect..

[8] Malfertheiner P., Megraud F., Rokkas T., Gisbert J.P., Liou J.-M., Schulz C., Gasbarrini A., Hunt R.H., Leja M., O’Morain C. (2022). Management of *Helicobacter pylori* infection: The Maastricht VI/Florence consensus report. Gut.

[9] Georgopoulos S.D., Michopoulos S., Rokkas T., Apostolopoulos P., Giamarellos E., Kamberoglou D., Mentis A., Triantafyllou K. (2020). Hellenic consensus on *Helicobacter pylori* infection. Ann. Gastroenterol..

[10] Chen M.J., Bair M., Chen P., Lee J., Yang T., Fang Y., Chen C., Chang A., Hsiao W., Yu J. (2022). Declining trends of prevalence of *Helicobacter pylori* infection and incidence of gastric cancer in Taiwan: An updated cross-sectional survey and meta-analysis. Helicobacter.

[11] Hooi J.K.Y., Lai W.Y., Ng W.K., Suen M.M.Y., Underwood F.E., Tanyingoh D., Malfertheiner P., Graham D.Y., Wong V.W.S., Wu J.C.Y. (2017). Global Prevalence of *Helicobacter pylori* Infection: Systematic Review and Meta-Analysis. Gastroenterology.

[12] Matos R., Taillieu E., De Bruyckere S., De Witte C., Rêma A., Santos-Sousa H., Nogueiro J., Reis C.A., Carneiro F., Haesebrouck F. (2022). Presence of *Helicobacter* Species in Gastric Mucosa of Human Patients and Outcome of Helicobacter Eradication Treatment. J. Pers. Med..

[13] Mladenova-Hristova I., Grekova O., Patel A. (2017). Zoonotic potential of *Helicobacter* spp. J. Microbiol. Immunol. Infect..

[14] Keikha M., Karbalaei M. (2022). Clinical aspects of *Helicobacter heilmannii*-associated gastritis in patients with dyspepsia: A systematic review and meta-analysis. Microb. Pathog..

[15] Matsumoto T., Kawakubo M., Akamatsu T., Koide N., Ogiwara N., Kubota S., Sugano M., Kawakami Y., Katsuyama T., Ota H. (2014). *Helicobacter heilmannii* sensu stricto-related gastric ulcers: A case report. World J. Gastroenterol..

[16] Yasuda T., Lee H.S., Nam S.Y., Katoh H., Ishibashi Y., Murayama S.Y., Matsui H., Masuda H., Rimbara E., Sakurazawa N. (2022). Non-*Helicobacter pylori Helicobacter* (NHPH) positive gastric cancer. Sci. Rep..

[17] Nishida R., Shimono N., Miyake N., Chong Y., Shimoda S., Tsukamoto H., Akashi K. (2017). *Helicobacter cinaedi* Bacteremia Mimicking a Flare of Systemic Lupus Erythematosus. Intern. Med..

[18] Baele M., Pasmans F., Flahou B., Chiers K., Ducatelle R., Haesebrouck F. (2009). Non-*Helicobacter pylori* helicobacters detected in the stomach of humans comprise several naturally occurring *Helicobacter* species in animals. FEMS Immunol. Med Microbiol..

[19] Taillieu E., De Witte C., De Schepper H., Van Moerkercke W., Rutten S., Michiels S., Arnst Y., De Bruyckere S., Francque S., van Aert F. (2023). Clinical significance and impact of gastric non-*Helicobacter pylori Helicobacter* species in gastric disease. Aliment. Pharmacol. Ther..

[20] Øverby A., Murayama S.Y., Michimae H., Suzuki H., Suzuki M., Serizawa H., Tamura R., Nakamura S., Takahashi S., Nakamura M. (2017). Prevalence of Gastric Non-*Helicobacter pylori*-Helicobacters in Japanese Patients with Gastric Disease. Digestion.

[21] Yakoob J., Abbas Z., Khan R., Naz S., Ahmad Z., Islam M., Awan S., Jafri F., Jafri W. (2012). Prevalence of non *Helicobacter pylori* species in patients presenting with dyspepsia. BMC Gastroenterol..

[22] Martinez-Gonzalez B., Moustakas N., Tsaoussi A., Nanou I., Michopoulos S., Georgopoulos S., Sgouras D.N. (2025). Non-*Helicobacter pylori Helicobacter* (NHPH) species in gastric biopsies from Greek symptomatic patients. Microb. Health Dis..

[23] Cortez Nunes F., Mateus T.L., Aguieiras C., Louro R., Peixe B., Calhindro M., Queirós P., Castelo-Branco P. (2025). Prevalence and Diagnostic Comparison of *Helicobacter pylori* and Non-*Helicobacter pylori Helicobacter* Infections in Patients Undergoing Upper Gastrointestinal Endoscopy with Gastric Biopsy in Algarve, Portugal. Microorganisms.

[24] Romo-Gonzalez C., Bustamante-Ogando J.C., Yamazaki-Nakashimada M.A., Aviles-Jimenez F., Otero-Mendoza F., Espinosa-Rosales F.J., Espinosa-Padilla S.E., Mendoza S.C.S., Durán-McKinster C., García-Romero M.T. (2021). Infections with Enterohepatic Non-*H. pylori Helicobacter* Species in X-Linked Agammaglobulinemia: Clinical Cases and Review of the Literature. Front. Cell. Infect. Microbiol..

[25] Flahou B., Haesebrouck F., Smet A., Yonezawa H., Osaki T., Kamiya S. (2013). Gastric and enterohepatic non-*Helicobacter pylori Helicobacters*. Helicobacter.

[26] Smet A., Menard A. (2020). Review: Other Helicobacter species. Helicobacter.

[27] Fox J.G., Chien C.C., Dewhirst F.E., Paster B.J., Shen Z., Melito P.L., Woodward D.L., Rodgers F.G. (2000). *Helicobacter canadensis* sp. nov. isolated from humans with diarrhea as an example of an emerging pathogen. J. Clin. Microbiol..

[28] Morgner A., Lehn N., Andersen L.P., Thiede C., Bennedsen M., Trebesius K., Neubauer B., Neubauer A., Stolte M., Bayerdörffer E. (2000). *Helicobacter heilmannii*-associated primary gastric low-grade MALT lymphoma: Complete remission after curing the infection. Gastroenterology.

[29] Cortez Nunes F., Teixeira S., Maia R.L., Amorim I., Mateus T.L. (2022). Perception and Knowledge of Portuguese Veterinarians about the Zoonotic Transmission of *Helicobacter pylori* and *Helicobacter suis*: The Need for One Health Intervention. Environ. Res. Public Health.

[30] Yamane T. (1967). Statistics: An Introductory Analysis.

[31] Schuppler M. (2025). On the Role of Food in the Transmission of *Helicobacter pylori* Infection: A Narrative Review. Foods.

[32] Joosten M., Flahou B., Meyns T., Smet A., Arts J., De Cooman L., Pasmans F., Ducatelle R., Haesebrouck F. (2013). Case report: *Helicobacter suis* infection in a pig veterinarian. Helicobacter.

[33] Angelidis S.A., Tirodimos I., Bobos M., Kalamaki M.S., Papageorgiou D.K., Arvanitidou M. (2011). Detection of *Helicobacter pylori* in raw bovine milk by fluorescence in situ hybridization (FISH). Int. J. Food Microbiol..

[34] Poutahidis T., Tsangaris T., Kanakoudis G., Vlemmas I., Iliadis N., Sofianou D. (2001). *Helicobacter pylori* -induced Gastritis in Experimentally Infected Conventional Piglets. Vet. Pathol..

[35] Bektas N., Besic L., Kulo Cesic A., Marjanovic D., Prguda-Mujic J. (2025). The first study on population knowledge and attitudes regarding prevention, diagnostic methods, treatment, and recovery aspects related to *Helicobacter pylori* infection in Bosnia and Herzegovina. Front. Public Health.

[36] Federation of Veterinarians of Europe https://fve.org/vetsurvey-is-now-available/.

[37] De Cooman L., Flahou B., Houf K., Smet A., Ducatelle R., Pasmans F., Haesebrouck F. (2013). Survival of *Helicobacter suis* bacteria in retail pig meat. Int. J. Food Microbiol..

[38] De Cooman L., Houf K., Smet A., Flahou B., Ducatelle R., De Bruyne E., Pasmans F., Haesebrouck F. (2014). Presence of *Helicobacter suis* on pork carcasses. Int. J. Food Microbiol..

[39] Mateus T.L., Teixeira P., Maia R.L., Leal Filho W., Vidal D.G., Dinis M.A.P., Dias R.C. (2022). We Know One Health, but We also Need One Communication. Sustainable Policies and Practices in Energy, Environment and Health Research.

[40] Skoufos S., Stavropoulou E., Tsigalou C., Voidarou C. (2025). Microbial Interconnections in One Health: A Critical Nexus Between Companion Animals and Human Microbiomes. Microorganisms.

